# Electrochemical Investigation of Oligonucleotide-DNA Hybridization on Poly(4-Methoxyphenethylamine)

**DOI:** 10.3390/ijms9071173

**Published:** 2008-07-08

**Authors:** Francielle B. Silva, Sabrina N. Vieira, Luiz R. Goulart Filho, Julien F. C. Boodts, Ana G. Brito-Madurro, João M. Madurro

**Affiliations:** 1 Institute of Chemistry, Federal University of Uberlândia, Uberlândia, 38400-902, Brazil; 2 Institute of Genetics and Biochemistry, Federal Univ. of Uberlândia, Uberlândia, 38400-902, Brazil

**Keywords:** electrochemical investigation, hybridization, immobilization, nucleotides, oligonucleotides, voltammetry

## Abstract

This work describes the immobilization of purine and pyrimidine bases and immobilization/hybridization of synthetic oligonucleotides on graphite electrodes modified with poly(4-methoxyphenethylamine) produced in acid medium. The immobilization of adenine, guanine, cytosine and thymine on these modified electrodes was efficient, producing characteristic peaks. Another relevant observation is that, according to the literature, pyrimidine bases, cytosine and thymine are more difficult to detect. However, when immobilized onto the poly(4-methoxyphenethylamine), a significant increase in the magnitude of the current was obtained. The observation of the hybridization between the poly(GA) probe and its complementary, poly(CT) target, was possible by monitoring the guanosine and adenosine peaks or through methylene blue indicator, using differential pulse voltammetry. Hybridization results in a decrease of the peak current of guanosine and adenosine or the signal of methylene blue accumulated on the modified electrode surface. The hybridization with the complementary target was also investigated by electrochemical impedance spectroscopy. The results showed a significant modification in the Nyquist plot, after addition of the complementary target, with increase of the charge transference resistance.

## 1. Introduction

Electrodes chemically modified by polymeric films have attracted considerable interest over the past two decades due to their potential application in electrochromic devices, electrocatalysis, electroanalysis and in the design of molecular electronic devices [1–4]. Electrochemical polymerization and precipitation of films directly onto an electrode surface offer some advantages when compared to the deposition of chemically prepared polymers, such as: simple preparation methodology, high reproducibility and thickness control.

The manipulation of the molecular composition of the electrode aims at improving sensitivity, selectivity and/or stability allowing for tailoring its response to meet analytical needs [3].

Electropolymerization of aromatic compounds has been investigated [5–9]. Previous studies indicate that monomers containing aromatic groups directly bonded to oxygen are easier to polymerize, present high reproducibility and mechanical resistance of the obtained film, allowing higher stability of the modified electrode [10–14]. For potential applications in catalysis, sensors and others, polymers functionalized with carboxyl, amino, aldehyde or succinimidyl carbonate groups favor the formation of covalent attachment with biomolecules.

The immobilization of biomolecular probes on an appropriate substrate is an important process since the sensitivity, the detection resolution and the reproducibility are significantly affected by the immobilization. In general, the surface of the substrate and/or terminus of DNA has been modified for stable immobilization of DNA probes [15].

Recently, we reported *in situ* preparation of electrodes coated with a conducting film of poly(4-aminophenol) [16, 17]. The results showed that this polymer prepared in acid medium has interesting properties, such as good stability and electrical conductivity. We observed also that graphite electrodes coated with this polymer were more efficient in immobilizing biomolecules when compared with non-coated graphite surfaces.

Electrochemical DNA sensing is a promising technique of nucleic acid analysis because of its speed, high sensitivity and low cost. This technique employs immobilized DNA sequences on the sensor surface as the recognition element and sequence-specific hybridization can be monitored and analyzed [18, 19].

Electrochemical methods [20–24] seem particularly well suited for the generally sample-limited case of DNA analysis, since they can be easily miniaturized (capable of working in nanoliter to picoliter volumes) without sacrificing sensitivity or selectivity. Up to date, most electrochemical detection protocols are based on the electroactivity of the nucleobases [25, 26].

Nucleic acid based biosensors, which are normally short synthetic oligonucleotides (ODNs), are progressively replacing genomic and cloned DNA because they are ideal chemical recognition elements, since hybridization is highly sequence-selective [27].

The use of oligonucleotides as probe and the appearance of a guanine signal upon hybridization with the target opened a new field in electrochemical research. This procedure eliminated the need for radioisotopic detection and shortened the assay time [28].

**Figure 1.**
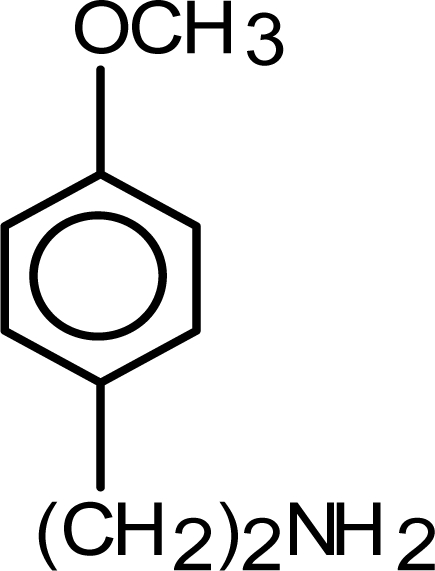
Structure of 4-methoxyphenethylamine.

In order to increase the DNA detection sensitivity we developed a new material based on electropolymerization of 4-methoxyphenethylamine (Figure 1) immobilized on a graphite surface.

With this aim, poly(4-methoxyphenethylamine) was synthesized and utilized for the immobilization of nitrogenated bases and oligonucleotide, investigated by differential pulse voltammetry. Electrochemical impedance spectroscopy was also used for to investigate of the hybridization of DNA complementary strand. Publications on the relations between electrochemical signals of solid electrodes, such as vitreous carbon, platinum, gold [16, 17, 27, 29, 30], and base sequences of poly- or oligonucleotides have appeared very rarely in the literature [31].

To our best knowledge, this is the first report on the eletrogeneration of poly(4-methoxyphenethylamine) and the application of this matrix for immobilization of nitrogenated bases and oligonucleotide and observation of complementary target.

## 2. Experimental

### 2.1. Chemicals

All reagents used were of analytical grade. Ultra high purity water (Millipore Milli-Q system) was used in the preparation of the solutions. Guanine 99+%, adenine 99.5+%, thymine 99+% and cytosine 99+% were purchased from Acros Organics and 4-methoxyphenethylamine ≥98% was purchased from Aldrich. The purine and pyrimidine concentrations were 20 mmol L^−1^ and 200 mmol L^−1^, respectively and prepared in water. The oligonucleotides were synthesized by Invitrogen Life Technologies with the following sequences: probe: poly(GA) 5′-GGG GGG GGA AAA AAA A-3′, complementary target: poly(CT) 3′-CCC CCC CCT TTT TTT T-5′. Stock solutions of the 6.4×10^−2^ mmol L^−1^ probe and 1.8×10^−1^ mmol L^−1^ target oligonucleotides were prepared in SSC 6X buffer (0.9 mol L^−1^ NaCl, 90 mmol L^−1^ sodium citrate, pH 7.0) and stored in a freezer until use. Buffer components (CH_3_COOH and CH_3_COONa or Na_2_HPO_4_ and NaH_2_PO_4_) were purchased from Sigma-Aldrich Chemical, USA (ACS purity) and prepared at pH 4.5 or pH 7.45 respectively. Monomer solutions were prepared in 0.5 mol L^−1^ HClO_4_ solution, immediately before their use. All reagents were used as received. The experiments were conducted at room temperature (25 ± 1°C).

### 2.2. Apparatus

Electrochemical polymerization and voltammetric measurements were performed in a three-compartment cell using a potentiostat from CH Instruments model 420A, with a graphite disk (6 mm diameter) cut from a graphite rod (99.9995%, Alfa Aesar) as working electrode. Platinum was used as counter electrode. All potentials are referred to the silver-silver chloride reference electrode (Ag/AgCl). The graphite surface, prior to electropolymerization, was mechanically polished with alumina slurry (0.3 μm diameter), ultrasonicated, washed with distilled water and dried in the air. All solutions were degassed by nitrogen bubbling.

### 2.3. Production of poly(4-methoxyphenethylamine) films

The electrochemical studies were performed in a three-compartment glass cell connected to a potentiostat. The monomer solutions were degassed with N_2_ prior to electropolymerization. Poly(4-methoxyphenethylamine) films were grown by potentiodynamic electropolymerization on graphite electrodes from 4-methoxyphenethylamine solution (15 mmol L^−1^). HClO_4_ solution (0.5 mol L^−1^) was used in all experiments.

### 2.4. Nitrogenated bases and oligonucleotide probe immobilization on graphite electrode/poly(4-methoxyphenethylamine)

The immobilization of biomolecules was carried out by appling 15 μL of stock solution of the 20 mmol L^−1^ purine and 200 mmol L^−1^ pyrimidine bases and 15 μL of 6.4×10^−2^ mmol L^−1^ poly(GA) probe to the modified electrode surface and dried at room temperature (25 ± 1°C) in a dessicator for 4 h. Then the electrode was immersed for 30 s in SSC 6X buffer. Differential pulse voltammetry measurements were conducted using acetate buffer (0.1 mol L^−1^, pH 4.5) or phosphate buffer (0.1 mol L^−1^, pH 7.45) as electrolyte.

### 2.5. Hybridization investigation of oligonucleotide immobilized onto poly(4-methoxyphenethylamine), using methylene blue as redox indicator or guanine and adenine monitoring

After the immobilization of the oligonucleotide poly(GA), 15 μL of 1.8×10^−1^ mmol L^−1^ poly(CT) (target) was applied to the modified electrode. Hybridization was carried out at 42°C for 15 minutes. The electrode was then rinsed by immersion in SSC 6X buffer during 30 s. Next, 15 μL of 20×10^−3^ mol. L^−1^ methylene blue in 20 × 10^−3^ mol L^−1^ NaCl was applied to the electrode surface. A final rinsing step was carried out by immersion in SSC 6X buffer during 30 s.

### 2.6. Impedance measurements

Graphite electrodes modified with poly(4-methoxyphenethylamine)/poly(GA) and poly(4-methoxyphenethylamine)/poly(GA):poly(CT) were investigated by electrochemical impedance spectroscopy.

Impedance measurements were carried with an Autolab PGSTAT 20 (Eco Chemie) equipment interfaced with a FRA module. The frequency interval investigated extended from 10^−3^ to 10^5^ Hz using a 5 mV (p/p) signal amplitude and logarithmic frequency scan approach.

The experimental data were fitted to a R_s_(Q_1_R_1_)(Q_2_[R_2_Z_T_]) equivalent circuit where R_s_ describes the solution resistance, R_ct_ the charge transfer resistance, Q_1_ and Q_2_ represent the double layer capacitance (due to the interface between the electrode and the electrolytic solution), Z_T_ represents the transmissive impedance. Studies were carried at a potential of +0.4V for electrode modified with polymeric film and + 0.9V for electrode modified with polymeric film with biomolecules immobilized at the surface.

## 3. Results and discussion

### 3.1. Electrochemical behavior of methoxyphenethylamine

**Figure 2.**
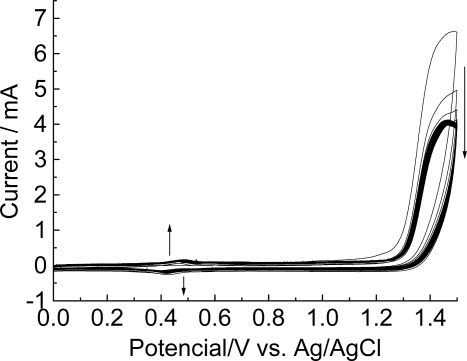
Cyclic voltammograms of graphite electrode in solution of 4-methoxyphenethylamine (15 mmol L^−1^) at pH 0.5; 50 mV.s^−1^, 100 scans. The arrows indicate the influence of the increasing number of scans.

Figure 2 shows the cyclic voltammetric behavior of 4-methoxyphenethylamine as function of continuous potential scanning.

As shown in Figure. 2, the potential cycle presents irreversible peaks at +1.48V that correspond to the formation of cation-radical intermediates of the monomer. During continuous potential cycling a gradual decrease of peak currents is observed. After the first cycle, a gradual increase in the peak current is observed between +0.3 and +0.6 V.

### 3.2. Electrochemical characterization of the modified electrodes

**Figure 3.**
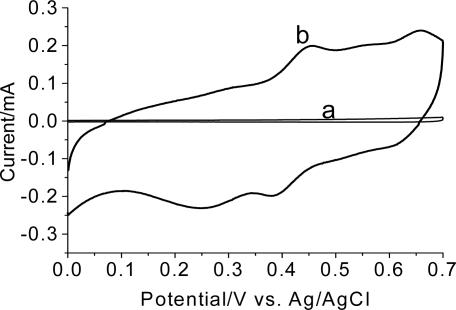
Cyclic voltammograms in HClO_4_ (0.5 mol L^−1^) of a bare graphite electrode (a) and the graphite electrode electrochemically treated (cyclic voltammetry between 0.0V to +1.50V vs. Ag/AgCl, 50 mV.s^−1^, 100 scans) in a 4-methoxyphenethylamine solution (15 mmol L^−1^) at pH 0.5 (b), 50 mV.s^−1^.

The electrochemical behavior of the graphite electrode electrochemically treated in a 4-methoxyphenethylamine solution (pH 0.5) is shown in Figure 3.

The main oxidation peaks of the graphite electrode modified in a solution of 4-methoxyphenethylamine are located at +0.45V and +0.65V while the reduction peaks are observed at +0.25V and +0.38V. This result indicates the formation of an electroactive polymer derived from 4-methoxyphenethylamine onto the graphite surface. The peaks separation does not mean that the electron transfer is irreversible but instead, is the consequence of a certain degree of resistance of the polymer as supported by the slopping current at the switching potential (E_λ_: +0.7V).

**Figure 4.**
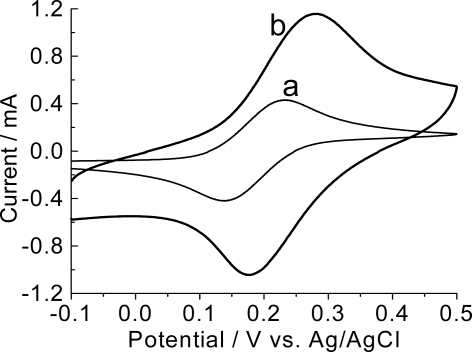
Cyclic voltammetric behavior of a bare graphite electrode (a) and a graphite electrode modified with poly(4-methoxyphenethylamine) (b) in aqueous solution containing K_3_Fe(CN)_6_ (5 mmol L^−1^)/K_4_Fe(CN)_6_ (5 mmol L^−1^) and KNO_3_ (0.1 mol L^−1^); 100 mV.s^−1^.

The cyclic voltammogram, in 0.1 mol L^−1^ KNO_3_ containing ferrocyanide/ferricyanide solution, of the electrode modified with poly(4-methoxyphenethylamine) is shown in Figure 4.

Formation of poly(4-methoxyphenethylamine), prepared in acid medium, presents an increase in the electrochemical response of the redox pair, when compared to the bare graphite electrode, indicating the modification of the graphite electrode, in agreement with the results of the modified electrode analyzed in HClO_4_ solution (Figure 3). A significant shift in the anodic peak potentials E_pa_, to more positive values, is observed, suggesting a slower electron transfer across the polymer.

### 3.3. Immobilization of purines and pyrimidines

**Figure 5.**
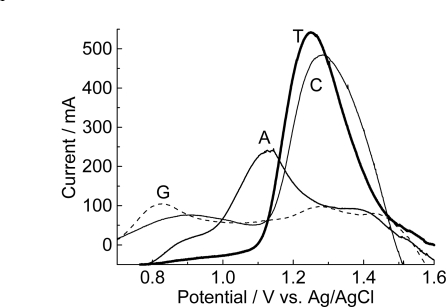
Differential pulse voltammograms baseline-corrected of graphite electrodes modified with poly(4-methoxyphenethylamine), prepared at pH 0.5, 100 scans, 15 μL of stock solution of each individual nitrogenated base applied. Concentration of stock solution: guanine, G, (20 mmol L^−1^), adenine, A, (20 mmol L^−1^), thymine, T, (200 mmol L^−1^) and cytosine, C, (200 mmol L^−1^). Electrolyte: 0.1 mol L^−1^ acetate buffer, pH 4.74. Modulation amplitude: 0.05mV. Pulse interval: 0.2s; 5mVs^−1^.

Purines: adenine and guanine, and pyrimidines, cytosine and thymine, were immobilized applying drops at room temperature onto the surface of modified electrodes prepared in acid pH (Figure 5).

Guanine, adenine, thymine and cytosine irreversibly oxidize on the modified graphite to produce oxidation peaks in acetate buffer.

Figure 5 shows that a response for all four nitrogenated bases, incorporated onto the surface of the graphite electrode modified with poly(4-methoxyphenethylamine), is observed.

The oxidation potentials of the immobilized bases onto the modified electrode are gathered in Table 1.

The pyrimidine bases, cytosine and thymine are more difficult to detect [27]. However, when immobilized onto the poly(4-methoxyphenethylamine) polymeric matrix, an increase in the magnitude of the current is obtained. This result is of interest for the analytical determination of DNA fragments with random amounts of pyrimidine and purine bases.

### 3.4. Investigation of the oligonucleotide immobilization

It is well known that large biomolecules can bind to polymers by adsorption [32]. Immobilization of oligonucleotide at solid supports present applications in forensic science, environmental studies, diagnosis and archeometry [33]. They progressively replace genomic and cloned DNA and are ideal chemical recognition elements, due to the fact that hybridization is a high sequence-selective feature [27].

**Figure 6.**
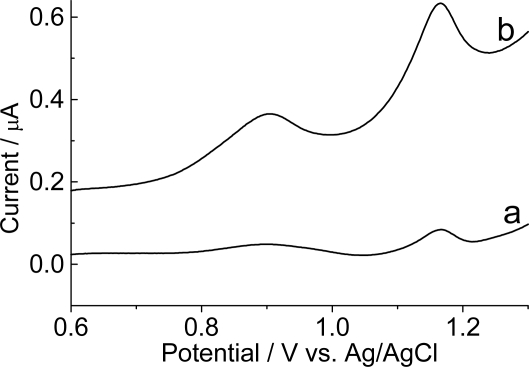
Differential pulse voltammograms of graphite electrode (baseline-corrected): (a) without film and (b) modified with poly(4-methoxyphenethylamine) containing 6.4×10^−2^ mmol L^−1^ of oligonucleotide probe, poly(GA). Electrolyte: 0.1mol L^−1^ phosphate buffer; pH 7.4, 100 mV s^−1^.

Heterooligomers of two DNA nucleobases present a significant electrochemical response. The immobilization of a 16-mer DNA sequence on a graphite electrode without film and after modification with poly(4-methoxyphenethylamine) is shown in Figure 6.

Figure 6 shows that the anodic peaks of purine oligonucleotides are located at potentials similar to those observed for the mononucleotides oxidation on graphite electrode modified with polymeric film (Figure 5). The electrodes modified with poly(4-methoxyphenethylamine) produced a current signal increase of the purine bases when compared to bare graphite electrodes, and produced oxidation peaks with good response and useful for detection of oligonucleotide. The magnitude of the current signal increased ca. 2.5 times for adenosine and guanosine.

**Figure 7.**
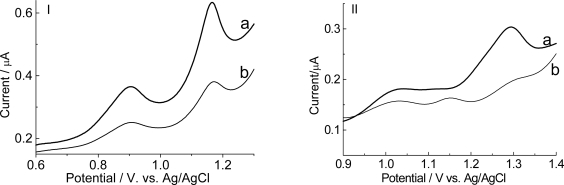
Differential pulse voltammograms of graphite electrode modified with poly(4-methoxyphenethylamine) prepared in pH 0.5 (baseline-corrected), 100 scans, containing poly(GA): (a) before hybridization and (b) after 20 minutes incubation with complementary target [poly(CT)]. Electrolyte: (I) 0.1mol L^−1^ phosphate buffer, pH 7.4; (II) 0.1mol L^−1^ acetate buffer, pH 4.74. Modulation amplitude: 0.05mV. Pulse interval: 0.2s; 5mVs^−1^.

The hybridization experiments were carried out in incubated solutions containing the complementary oligonucleotide. Figure 7 presents the differential pulse response recorded in phosphate buffer or acetate buffer as electrolyte.

The peak currents of guanosine and adenine gradually decreased after 15 minutes of incubation in phosphate or acetate buffer, in agreement with Oliveira-Brett and col. [27], who reported that during the oligonucleotides hybridization, hydrogen bonds are formed between complementary sequences leading to a duplex, inside of which it is more difficult to oxidize the bases, decreasing the oxidation peak current of the guanosine and adenosine, after hybridization.

**Figure 8.**
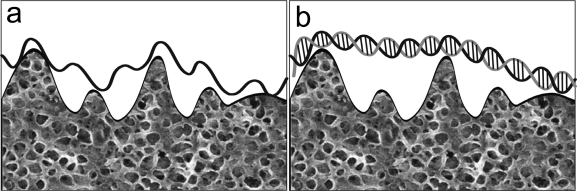
Immobilization of oligonucleotide onto the surface of the modified electrode: (a) before or (b) after hybridization or duplex formation.

Another reason to the higher current values obtained for single strand DNA is that the latter presents higher proximity and a higher degree of adsorption onto the electrode surface, due to its higher conformational flexibility (Figure 8), facilitating the charge transfer between the nitrogenated bases and the electrode [34, 35].

**Figure 9.**
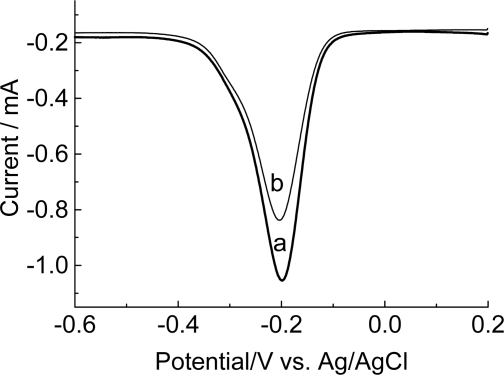
Differential pulse voltammograms of accumulated methylene blue on the electrode modified with polymeric film/poly(GA) (baseline-corrected) before (a) or after (b) hybridization with the complementary poly(CT) target oligonucleotide. Electrolyte: 0.1mol L^−1^ phosphate buffer, pH 7.4. Modulation amplitude: 0.05mV. Pulse interval: 0.2s; 5mVs^−1^.

Others experiments were carried out using an electrode modified with film/poly(GA) and an electrode modified with film/poly(GA):poly(CT) as transducers and methylene blue as electroactive indicator (Figure 9).

Methylene blue interacts in different forms with the simple strand DNA and the double strand DNA, therefore governing its signal of reduction. This fact reflects the hybridization extension with the immobilized probe [36]. Evidences of the direct interaction of methylene blue with guanine have been reported by various authors [37–39].

**Figure 10.**
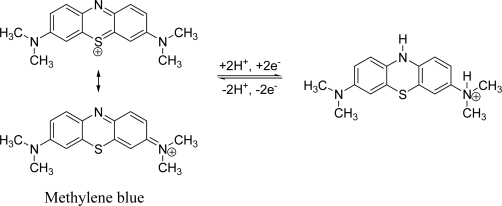
Methylene blue reduction at a solid substrate [37].

The decrease in the values of the reduction current is governed by the specific interactions between methylene blue and free guanine, with lesser accessibility of methylene blue to guanine in hybridized DNA [40]. The electron transfer between the electrode and the indicator (methylene blue) is in this form, substantially modified due to the smaller amount of species in the presence of the double strand DNA passing from the oxidized state to the reduced state, Figure 10 [37]. This fact is in agreement with the obtained results (see Figure 9).

The hybridization effect was also characterized using other techniques besides cyclic and differential pulse voltammetry. Electrochemical impedance spectroscopy (EIS) is proposed as the transduction principle for the detection of DNA complementary strands [41].

**Figure 11.**
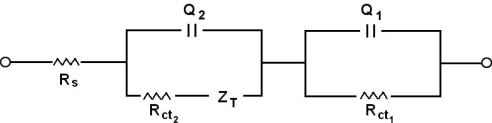
Equivalent electrical circuit used to fit the experimental impedance data. R_s_ describes the solution resistance, R_ct_ the charge transfer resistances, Q_1_ and Q_2_ represent constant phase elements, Z_T_ represents the transmissive impedance.

Figure 11 shows the equivalent circuit, EC, used to fit the experimental results. For the poly(4-methoxyphenethylamine)/poly(GA) system there was no need for the Z_T_ component.

**Figure 12.**
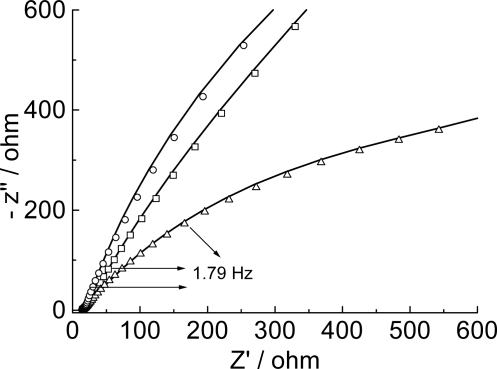
Nyquist diagram for EIS measurements in 0.1 mol L^−1^ phosphate buffer for modified graphite electrode with film. (Δ) Without biomolecules; after poly(GA) probe–modification: without (○) and with (□) hybridization with poly(CT) target. The continuous lines represent the fitting to the equivalent circuit shown in Figure 11.

Figure 12 presents the complex plane plot representative of the impedance response of a graphite electrode modified with the film and film/poly(GA) or film poly(GA):poly(CT). Impedance spectra were recorded using next experimental conditions: +0.4V for the graphite electrode modified with polymeric film and +0.9V for the graphite electrode modified with polymeric films with immobilized biomolecules, using as electrolyte 0.1 mol L^−1^ phosphate buffer.

Table 2 gathers the fitting results.

The chi-square values of the Kramers-Kronig test of the order of 10^−2^–10^−3^ attest the good quality of the data. Q is defined as the double layer capacitance, Y_0_ is defined as the Q absolute value, *n*_1_, *n*_2_ and B is the phase angle displacement.

Some interesting features came out of the data gathered in table 2. The solution resistance (R_s_) is reasonably constant, centered around 14.3 ± 1.3. This is a reasonable value considering the electrolyte composition. The slight dispersion of the R_s_–values is acceptable and is obviously due to the difficulty to position the electrodes at exactly the same distance between experiments.

Two (QR) serial combinations, with an additional T component for the film/poly(GA):poly(CT) system, were required to fit the results, suggesting that two different film regions contribute to the impedance response. To take into account the frequency dispersion, due to roughness/porosity of the systems, it was necessary to use two constant phase elements, Q, instead of pure capacitor elements. However, the n_1_-values close 0.5 of the Q_1_R_1_ combination supports Q_1_ behaves very similarly to a faradaic behavior. The n_2_-values values present values between 0.5 and 1.0 suggesting a behavior between capacitance and Warburg.

Comparison of the Q_1_ and Q_2_ values for the film+poly(GA) and film+poly(GA):poly(CT) with the data for the film supports an increase in the surface area after the application of poly(GA) and poly(GA):poly(CT). The application of the latter, however, results in approximately 10 to 15× higher enhancement. This is a reasonable result. The model for the morphology of the electroactive layer that emerges from the results supports the existence of two regions: a more internal region, close to the graphite surface, described by the R_1_ and Q_1_ data, and a more external region after the poly(CT) is applied described by the Q_2_ R_2_ data. The modest increase in Q_1_ is due the presence of poly(GA) and its rather similar value with the Q_1_-value for poly(GA):poly(CT) suggests that the poly(GA) strand is adsorbed closely to but not parallel to the graphite surface. The significant increase of Q_2_ compared to Q_1_ suggests that the CT strand is adsorbed, with its elements in close contact with the poly(GA) strand (hybridization), increasing the distance of the double strand formed with the electrode surface, due to the lower conformational flexibility, resulting in a decrease of the charge transfer (see Figure 8). This conformational model is consistent with R_ct_-values. In fact, for the more internal region electron transfer is expected to be easier resulting in lower R^1^ct-values when compared to the R^2^ct-values. The latter values are indeed about 600–800 times higher. The introduction of poly(CT) increases the R^2^ct compared to the R^1^ct-value, consistent with the oxidation of more distant oxidable sites.

## 4. Conclusions

The data presented in this work showed that poly(4-methoxyphenethylamine) is an efficient matrix for purines and pyrimidines immobilization. Differential pulse voltammetry and electrochemical impedance spectroscopy have been used to characterize the immobilization and hybridization process of nitrogenated bases and/or oligonucleotide.

All DNA bases are electroactive and give a significant electrochemical response on modified electrodes, in the absence (adenine, guanine, thymine and cytosine) or in the presence (oligonucleotide) of phosphate groups onto a graphite electrode modified with polymeric films.

The results support the oligonucleotide hybridization with the complementary target. Additionally, the interaction between DNA molecules and small biomolecules (methylene blue) can be observed through differential pulse voltammetry and electrochemical impedance spectroscopy. Based on the response of the indicator, the complementary DNA sequence produces a decrease in the current signal, result in agreement with the literature. The oligonucleotide hybridization with the complementary target, analyzed by electrochemical impedance spectroscopy, showed a significant modification in the Nyquist plot upon addition of the complementary target with increase of the charge transference resistance.

The combination of a graphite electrode with poly(4-methoxyphenethylamine) is a promising strategy for the DNA probe immobilization and other biological recognition elements.

## Figures and Tables

**Table 1.** Oxidation potential of individual nitrogenated bases immobilized onto the electrodes modified with poly(4-methoxyphenethylamine). Electrolyte: acetate buffer.

Guanine		Adenine		Thymine		Cytosine	
Potential (V)	Current (mA)	Potential (V)	Current (mA)	Potential (V)	Current (mA)	Potential (V)	Current (mA)
0.829	103	1.132	236	1.250	541	1.277	482

**Table 2.** The fitting values of the equivalent circuit elements.

	R_S_(′Ω)	Q_1_		R^1^_ct_(ohm)	Q_2_		R^2^(ohm)	Z_T_	
		Y_0_(μF)	*n*_1_		Y_0_(mF)	*n*_2_		Y_0_	B
Film	15.7	13.4	0.62	875	0.1250	0.736	326	0.0200	0.201
Film/Poly(GA)	13.3	36.8	0.46	5.55	0.5401	0.848	3330	-	-
Filme/Poly(GA): Poly(CT)	14.0	32.0	0.54	5.16	0.1080	0.762	4400	0.003564	0.177

## References

[1] Banks CE, Davies TJ, Wildgoose GG, Compton RG (2005). Electrocatalysis at Graphite and Carbon Nanotube Modified Electrodes: Edge-Plane Sites and Tube Ends Are the Reactive Sites. Chem Commun.

[2] Blanco-Lopez MC, Lobo-Castanon MJ, Miranda-Ordieres AJ, Tunon-Blanco P (2003). Voltammetric Sensor for Vanillylmandelic Acid Based on Molecularly Imprinted Polymer-Modified Electrodes. Biosens Bioelectron.

[3] Kutner W, Wang J, L'her M, Buck R (1998). Analytical Aspects of Chemically Modified Electrodes: Classification, Critical Evaluation and Recommendations. Pure Appl Chem.

[4] Chidsey CED, Murray RW (1986). Electroactive Polymers and Macromolecular Electronics. Science.

[5] Bardavid Y, Kotlyar AB, Yitzchaik S (2006). Conducting Polymers Coated DNA. Macromol Symp.

[6] Kulkarni MV, Viswanath AK, Khanna PK (2006). Synthesis and Characterization of Poly(N-Methyl Aniline) Doped with Sulphonic Acids: Their Application as Humidity Sensors. J Appl Polym Sci.

[7] Menezes HA, Maia G (2006). Films Formed by the Electrooxidation of *p*-Aminophenol (*p*-Aph) in Aqueous Medium: What Do They Look Like?. J Electroanal Chem.

[8] Topcu-Sulak M, Gokdogan O, Gulce A, Gulce H (2006). Amperometric Glucose Biosensor Based on Gold-Deposited Polyvinylferrocene Film on Pt Electrode. Biosens Bioelectron.

[9] Ortega JM (2000). Electrodeposition of Copper on Poly (*o*-Aminophenol) Modified Platinum Electrode. Thin Solid Films.

[10] Lofrano RCZ, Madurro JM, Abrantes LM, Romero JR (2004). Electrocatalytic Hydrogenation of Carbonylic Compounds Using an Electrode with Platinum Particles Dispersed in Films of Poly-[Allyl Ether P-(2-Aminoethyl) Phenol]. J Mol Catal A: Chem.

[11] Lofrano RCZ, Madurro JM, Romero JR (2000). Preparation and Properties of an Electrode Coated with a Cerium Poly(Allyl Ether *p*-Benzenesulfonate) Film for Electrorganic Reactions. J Mol Catal A: Chem.

[12] Castro CM, Vieira SN, Goncalves RA, Madurro AGB, Madurro JM (2008). Electrochemical and Morphologic Studies of Nickel Incorporation on Graphite Electrodes Modified with Polytyramine. J Mater Sci.

[13] Franco DL, Afonso AS, Vieira SN, Ferreira LF, Goncalves RA, Madurro AGB, Madurro JM (2008). Electropolymerization of 3-Aminophenol on Carbon Graphite Surface: Electric and Morphologic Properties. Mater Chem Phys.

[14] Vieira SN, Ferreira LF, Franco DL, Afonso AS, Goncalves RA, Madurro AGB, Madurro JM (2006). Electrochemical Modification of Graphite Electrodes with Poly(4-Aminophenol). Macromol Symp.

[15] Rho S, Jahng D, Lim JH, Choi J, Chang JH, Lee SC, Kim KJ (2008). Electrochemical DNA Biosensors Based on Thin Gold Films Sputtered on Capacitive Nanoporous Niobium Oxide. Biosens Bioelectron.

[16] Ferreira LF, Boodts JFC, Madurro AGB, Madurro JM (2008). Gold Electrodes Modified with Poly(4-Aminophenol): Incorporation of Nitrogenated Bases and an Oligonucleotide. Polym Int.

[17] Madurro AGB, Ferreira LF, Vieira SN, Goulart LR, Madurro JM (2007). Immobilization of Purine Bases in Poly-4-Aminophenol Matrix. J Mater Sci.

[18] Popovich ND, Eckhardt AE, Mikulecky JC, Napier ME, Thomas RS (2002). Electrochemical Sensor for Detection of Unmodified Nucleic Acids. Talanta.

[19] Wang J, Palecek E, Nielsen PE, Rivas G, Cai X, Shiraishi H, Dontha N, Luo D, Farias PAM (1996). Peptide Nucleic Acid Probes for Sequence-Specific DNA Biosensors. J Am Chem Soc.

[20] Xu H, Liu X, Cui D, Li M, Jiang M (2006). A Novel Method for Improving the Performance of ZnO Gas Sensors. Sens Actuators B.

[21] Palecek E (1996). From Polarography of DNA to Microanalysis with Nucleic Acid-Modified Electrodes. Electroanalysis.

[22] Mikkelson SR (1996). Electrochemical Biosensors for DNA Sequence Detection. Electroanalysis.

[23] Palecek E, Postbieglova I (1986). Adsorptive Stripping Voltammetry of Biomacromolecules with Transfer of the Adsorbed Layer. J Electroanal Chem.

[24] Palecek E (1960). Oscillographic Polarography of Highly Polymerized Deoxyribonucleic Acid. Nature.

[25] Lin H, Xu DK, Chen HY (1997). Simultaneous Determination of Purine Bases, Ribonucleosides and Ribonucleotides by Capillary Electrophoresis-Electrochemistry with a Copper Electrode. J Chromatogr.

[26] Kafil JB, Cheng HY, Last T (1986). Quantitation of Nucleic Acids at the Picogram Level Using HPLC with Electrochemical Detection. Anal Chem.

[27] Oliveira-Brett AM, Piedade JA, Silva LA, Diculescu VC (2004). Voltammetric Determination of all DNA Nucleotides. Anal Biochem.

[28] Ozkan D, Erdem A, Kara P, Kerman K, Meric B, Hassmann J, Ozsoz M (2002). Allele-Specific Genotype Detection of Factor V Leiden Mutation from Polymerase Chain Reaction Amplicons Based on Label-Free Electrochemical Genosensor. Anal Chem.

[29] Lassalle N, Vieil E, Correia JP, Abrantes LM (2001). Study of DNA Hybridization on Polypyrrole Grafted with Oligonucleotides by Photocurrent Spectroscopy. Biosens Bioelectron.

[30] Franco DL, Afonso AS, Ferreira LF, Goncalves RA, Boodts JFC, Madurro AGB, Madurro JM (2008). Electrodes Modified with Polyaminophenols: Immobilization of Purines and Pyrimidines. Pol Eng Sci.

[31] Stempkowska I, Ligaj M, Jasnowska J, Langer J, Filipiak M (2007). Electrochemical Response of Oligonucleotides on Carbon Paste Electrode. Bioelectrochemistry.

[32] Pham MC, Piro B, Tran LD (2003). Direct Electrochemical Detection of Oligonucleotide Hybridization on Poly(5-Hydroxy-1,4-Naphthoquinone-*Co*-5-Hydroxy-3-Thioacetic Acid-1,4-Naphthoquinone) Film. Anal Chem.

[33] Okutucu B, Telefoncu A (2004). Covalent Attachment of Oligonucleotides to Cellulose Acetate Membranes. Artif Cells Blood Substit Immobil Biotechnol.

[34] Yang M, McGovern ME, Thompson M (1997). Genosensor Technology and the Detection of Interfacial Nucleic Acid Chemistry. Anal Chim Acta.

[35] La-Scalea MA, Serrano SHP, Gutz IGR (1999). Eletrodos Modificados com DNA: uma Nova Alternativa em Eletroanálise. Quím Nova.

[36] Jin Y, Yao X, Liu Q, Li J (2007). Hairpin DNA Probe Based Electrochemical Biosensor Using Methylene Blue as Hybridization Indicator. Biosens Bioelectron.

[37] Yan F, Erdem A, Meric B, Kerman K, Ozsoz M (2001). Electrochemical DNA Biosensor for the Detection of Specific Gene Related to Microcystis Species. Electrochem Commum.

[38] Rohs R, Sklenar H, Lavery R, Roder B (2000). Methylene Blue Binding to DNA with Alternating GC Base Sequence: a Modeling Study. J Am Chem Soc.

[39] Enescu M, Bernard L, Gheorghe V (2000). Molecular Dynamics Simulation of Methylene Blue – Guanine Complex in Water: the Role of Solvent in Stacking. J Phys Chem.

[40] Kara P, Kermam K, Ozkan D, Meric B, Erdem A, Ozkan Z, Ozsoz M (2002). Electrochemical Genosensor for the Detection of Interaction Between Methylene Blue and DNA. Electrochem Commun.

[41] Bonanni A, Esplandiu MJ, Pividori MI, Alegret S, del Valle M (2006). Impedimetric Genosensors for the Detection of DNA Hybridization. Anal Bioanal Chem.

